# Interventions to improve quality of life (QOL) and/or mood in patients with head and neck cancer (HNC): a review of the evidence

**DOI:** 10.1186/s41199-019-0041-4

**Published:** 2019-06-11

**Authors:** Jordan J. Senchak, Carolyn Y. Fang, Jessica R. Bauman

**Affiliations:** 1 Department of Internal Medicine, Temple University Hospital, 1801 N. Broad Street, Philadelphia, PA 19122 USA; 2 Cancer Prevention and Control Program, Fox Chase Cancer Center, 333 Cottman Avenue, Philadelphia, PA 19111 USA; 3 Department of Hematology/Oncology, Fox Chase Cancer Center, 333 Cottman Avenue, Philadelphia, PA 19111 USA

**Keywords:** Head and neck cancer (HNC), Quality of life (QOL), Interventions, Psychological distress, Mood, Interventions

## Abstract

**Background:**

Patients with head and neck cancer (HNC) experience significant physical and psychological distress, which have a negative impact on their quality of life (QOL). Few strategies have been studied to help improve QOL in this patient population.

**Results:**

In this article, we review the existing literature for intervention studies that focus on improving QOL and/or mood in HNC patients. Our review yielded 14 studies that met criteria. Types of interventions included educational, psychosocial, physical and psychological symptom management, mindfulness, pharmacologic, exercise, and telemedicine. Although the majority of the studies had small sample sizes or other methodological limitations, many showed preliminary feasibility and acceptability with some positive impacts on QOL and/or mood.

**Conclusions:**

Larger studies are warranted with more robust randomized designs to determine efficacy of interventions to improve QOL and/or mood in patients with HNC. Additionally, future studies must also consider strategies for implementation and dissemination of these interventions into the health care system to improve the physical and psychological burden of HNC as a population.

## Background

Patients with head and neck cancer (HNC) can often be cured with cancer treatment. However, patients and their caregivers report many unmet needs throughout treatment, recovery, and survivorship that affect overall quality of life (QOL) and mood. Treatment for HNC is complex, involving intricate surgery, radiation, and/or chemotherapy, which together offer unique challenges for these patients. Specifically, treatment involves changes to critical structures for speaking, eating, and breathing, which can lead to functionality issues such as dysphagia and breathing difficulties, as well as a cosmetic burden with facial and neck disfigurement [1]. Additionally, the symptom burden during treatment is significant as patients report a myriad of symptoms including severe pain, distress, dehydration, malnutrition, nausea, constipation, and sleep disturbances [2, 3].

Throughout this intense treatment, psychological distress is particularly high in patients with HNC, with levels of depression, anxiety, and post-traumatic stress disorder (PTSD) among the highest reported of any cancer population [4, 5]. Between 15 and 50% of HNC patients are affected by major depressive disorder at some point during their treatment [5]. Depression levels change over the treatment trajectory, with one study showing the highest levels at the completion of radiotherapy [6]. Suicide rates are four times higher than the general population [7]. Patients who experience higher levels of depression during treatment can face worse outcomes than those who experience lower levels of depression during treatment [4]. Patients with HNC and untreated psychological needs have a worse QOL, longer hospital stays, worse post-operative performance status, increased narcotic use, decreased treatment compliance, and increased complications with treatment [8]. Additionally, numerous studies demonstrate a link between depression and worse survival [9, 10].

Not only is depression a major problem for patients with HNC, but those undergoing treatment also experience significant levels of anxiety and PTSD [4, 11]. Gil et al noted that patients often experience the highest levels of anxiety at the time of diagnosis^12^. Additionally, in the face of a new diagnosis, 12% of patients and 29% of caregivers met criteria for PTSD [11]. HNC patients often deal with cosmetic issues due to radiation and surgical interventions. This lasting disfigurement increases the risk of PTSD [12, 13]. In a study by Moschopoulou et al, the prevalence of PTSD at the 6-year mark was found to be 13%, which is significantly higher than the general population [12]. Thus, improving how we address psychological symptoms is paramount to patients’ overall QOL and mood as we strive to optimize the care of our patients with HNC.

Among cancer survivors, evidence-based interventions have proven effective in reducing psychological distress associated with a cancer diagnosis or treatment and improving QOL [14]. However, few such interventions have been designed and implemented to ameliorate the severe psychological and functional burden of HNC. Thus, the primary objective of this paper is to review these interventions and evaluate their impact on psychological symptoms and QOL, with a particular focus on study methodology, outcome measures, and efficacy. We conclude with a summary of the methodological challenges in these trials and propose future research directions to improve the QOL and mood of patients with HNC.

## Methods

### Search and selection criteria

We conducted a literature search using the online PubMed database to identify studies in English through June 2018 of interventions during or after HNC treatment designed to improve psychological symptoms and/or QOL in patients as either a primary or secondary endpoint. Authors JS and JB performed this search independently and then compared results, deciding together whether to include each study. Key words and phrases used alone and/or in combination for the search included HNC, QOL, intervention, depression, anxiety, PTSD, and/or randomized-controlled trial (RCT). Reference lists from citations were also reviewed for relevant publications. We included studies with a prospective intervention to improve QOL and or psychological symptoms during or after HNC treatment. Studies could be RCTs, non-randomized, controlled trials, or pilot studies. We excluded studies that were not prospective interventions (1 study), those that were ongoing or in development without outcomes reported (3 studies), or those that did not collect patient reported outcomes (1 study). Using the designated search strategy, 14 studies were identified. The methodological and statistical quality of the studies was assessed using a 7-item checklist with the following criteria: (A) sample characteristics, (B) Sample size, (C) Data collection, (D) Response Rates, (E) Outcome measurement, (F) Comparison groups, (G) Statistical Analyses adapted from previous published, standardized checklists [15]. Descriptive statistics were calculated using Microsoft Excel. Authors JS and JB scored all 14 studies independently and then discussed discrepancies until a consensus was formed.

### Study characteristics

Our search identified 14 studies that met the criteria [16–29]. Tables 1, 2, 3 and 4 list these studies with a summary of their patient populations, interventions and timing of interventions in relation to cancer treatment, outcome measures assessed and timing of administration, results, and quality assessment. The interventions are categorized based on the type of intervention: nurse-led interventions in Table 1 [16–19], psychologist-led interventions in Table 2 [20–23, 28], pharmacologic and lifestyle interventions in Table 3 [24, 25], and health systems interventions in Table 4 [26, 27]. Of the 14 studies, 7 were RCTs [16, 17, 19, 22–25], 3 were non-randomized, controlled trials [18, 26, 27], and 4 were single arm studies with no control group [20, 21, 28, 29]. The interventions were diverse in content and included educational, psychosocial, physical and psychological symptom management, mindfulness, pharmacologic, exercise, and telemedicine. Six of the interventions were delivered from the time of diagnosis and during cancer treatment [16, 22, 24, 27–29], four were delivered after cancer treatment ended [18, 19, 23, 26], three included mixed populations of patients during and after cancer treatment ended [17, 20, 25], and one study [21] gave no information on where patients were in the treatment trajectory. Four studies enrolled only patients with psychosocial or psychological dysfunction as part of entry criteria [17, 18, 22, 23]. The outcomes of QOL, anxiety, depression, and PTSD were measured using multiple different assessment tools. For QOL, measures included the European Organization of Research and Treatment of Cancer Quality of Life Questionnaire (EORTC-QOL) [16, 20, 21, 23], Functional Assessment of Cancer Therapy (FACT) [25, 28, 29], and/or the University of Washington Quality of Life (UWQOL) [18, 24]. Depression and/or anxiety were measured using the Hospital Anxiety and Depression Scale (HADS) [18, 20, 23, 27], State Trait Anxiety Inventory [16, 22], Quick Inventory of Depressive Symptomatology (QIDS) [24], Geriatric Depression Scale-Short Form [17], Beck Depression Inventory [22], and/or Center for Epidemiological Studies-Depression Score (CES-D) [19, 21]. One study measured PTSD using the Clinician Administered PTSD scale [22]. The studies were of varying quality (range 3–7, median 5.5). Only 7/14 (50%) had adequate sample size, and only 4/14 (29%) had participation and response rates above 75%. Otherwise, sample characteristics, data collection, outcome measures, and statistical analyses were all well described and standardized. Below we summarize each subgroup of interventions and highlight relevant findings and key methodological issues that emerged.

**Table 1:** Nurse-delivered interventions

Study	Settings/Patients	Study Design/Intervention	Timing of intervention	Assessment timepoints	Measures	Results	Methodological Quality^a^
Katz et al. (2004) [16]	19 HNC pts. (10 experimental, 9 control); Canada	RCT. Intervention group received 2 sessions with research nurse (60-90 min each) with educational booklet covering info on diagnosis and surgery, what to expect physically post-surgery, effective coping strategies.	Newly diagnosed oral cavity cancer pre-treatment; sessions were pre-operatively then pre-discharge from hospital	baseline, pre-hospital discharge, 3 months	Knowledge, State-trait anxiety inventory (STAI), CES-D, affect balance scale, Atkinson Life Happiness rating scale, illness intrusiveness ratings scale, EORTC QLQ-30; satisfaction with intervention survey	Experimental group showed gain in knowledge, less body image disturbance, lower anxiety	5
Duffy et al. (2006) [17]	184 HNC pts. randomized; pts. had at least one of the following: smoking, alcohol use, or depression; Michigan	RCT. Intervention group received sessions with a nurse consisting of cognitive behavioral therapy with workbook and phone calls plus medications as needed for smoking cessation or depression.	Any time after diagnosis (0 to 282 months; mean 24 months from diagnosis)	baseline and 6 months	Alcohol use: Alcohol Use Disorder Identification Test; Depression: Geriatric Depression Scale-Short Form	Significant differences in 6 month smoking cessation rates. No significant difference in 6 month depression/alcohol use.	7
Semple et al. (2009) [18]	54 HNC pts., meeting cutoffs for psychosocial dysfunction (hospital depression scale, work and social adjustment scale-WASA); Ireland	Non-randomized, controlled trial. Intervention group was self-selected and received one-on-one home visits with clinical nurse specialist for an individualized, problem-focused program with bibliotherapy as adjunct. Controls were those who self-selected not to participate.	Post-treatment (no time frame given, no details of treatment), 2–6 home sessions (avg 4), up to 90 min each with 2 weeks between sessions	baseline, 1 week post intervention, 3 months	Psychological dysfunction: HADS; WASA and QOL: HRQOL by UWQOL v. 4; Work and Social Adjustment Scale	Significant improvement in psychological distress, social functioning and some QOL scores, sustained in 3 month follow up period	6
van der Meulen et al. (2014) [19]	205 HNC pts. HNC cancer	RCT. Intervention group received 6 bimonthly, problem focused counseling sessions (45 min each) with a nurse addressing physical, psychological, and social consequences of HNC.	Enrolled before treatment started but sessions started after treatment ended	baseline, 3, 6, 9, and 12 months after treatment completion	Depression: CES-D; QOL: EORTC QLQ	Depression levels were significantly lower in the intervention group at 1 year and physical symptoms also decreased in the intervention group compared to control	6

^a^Criteria for assessing the methodological and statistical quality of the studies were adapted from Longacre et al., where one point was given for each criteria met of the following categories: (A) Sample characteristics, (B) Sample size, (C) Data collection, (D) Response rates, (E) Outcome measurement, (F) Comparison groups, and (G) Statistical analyses. A total of 7 points was possible such that higher points were higher quality studies

**Table 2:** Psychologist-delivered interventions

Study	Settings/Patients	Study Design/Intervention	Timing of intervention	Assessment timepoints	Measures	Results	Methodological Quality
Hammerlid et al. (1999) [20]	13 HNC pts. in Sweden	Single arm studies. Two studies: first, long term psychological group therapy for patients with newly diagnosed HNC with psychologist. Second, 1 week long psychoeducational program 1 year after treatment for HNC with oncologist, nurse, and physiotherapist.	1. Started with new diagnosis of HNC; 2. Started 1 year after treatment	baseline, 1, 2, 3, 6, and 12 months	HADS; EORTC; study-specific questionnaire	QOL in therapy group improved more than control	3
Allison PJ et al. (2004) [21]	50 HNC pts.; Canada	Single arm study. Intervention group received Nucare coping strategies, psychoeducational intervention that teaches coping in one of three formats (small group with therapist, one on one with therapist, or a home format without therapist)	no information about where patients were in treatment or type of treatment or subtype of HNC	baseline and 3 months	EORTC-QOL; HADS	Intervention resulted in higher health related QOL and depression scores.	5
Kangas et al. (2013) [22]	35 HNC pts. with elevated levels of PTSD, depression, or anxiety	RCT. Intervention group received seven weekly individual sessions with clinical psychologist of multi-modal CBT vs non-directive supportive counseling, concurrent with patient’s radiotherapy.	concurrent with radiotherapy	baseline, 1, 6, and 12 months	Clinician Administered PTSD Scale; Beck Depression Inventory; State Trait Anxiety Inventory; FACT-General	CBT and SC interventions found to be equal in effects reducing PTSD and anxiety symptoms in short/long term. However, more pts. in CBT program no longer met clinical or sub clinical PTSD, anxiety, and/or depression by 12 months post treatment	5
Kilbourn et al. (2013) [28]	24 HNC pts.	Single arm study. Easing and Alleviating Symptoms during Treatment (EASE) intervention – participants received up to 8 telephone counseling sessions focused on coping and stress management.	Concurrent with radiotherapy	Baseline, 1 month post intervention end	Impact of Events Scale, FACT-HN, Pain Disability Index, Interpersonal Support Evaluation List	Intervention was feasible and acceptable. Participants experienced decrease in QOL and no change in pain scores.	4
Krebber et al. (2016) [23]	156 pts. (HNC and lung cancer (LC)); had distress on HADS to be included; Netherlands	RCT. Intervention group received stepped care, which consisted of watchful waiting, guided self-help, problem-solving therapy, and psychotherapy and/or psychotropic medication with oncologist or psychologist/psychiatrist as stepped care.	enrolled within 1 month of completing curative treatment for LC or HNC (94%), no specifics on surgery vs. RT vs CRT	baseline, completion of care, 3, 6, 9, 12 months	HADS; EORTC QLQ-C30, QLQ-HN35/QLQ-LC13, IN-PATSAT32 (satisfaction with care)	Psychological distress better in the stepped care group vs usual care. Those patients with anxiety or depression had an even larger improvement in the stepped care group.	6
Pollard et al. (2017) [29]	19 HNC pts.	Single arm study. Mindfulness intervention – participants received 7, 90 min one-on-one sessions with clinical psychologist with individualized mindfulness-based stress reduction (IMBSR) program.	Concurrent with radiotherapy	Baseline and post-intervention/ treatment	Five-Factor Mindfulness Questionnaire (FFMQ), Profile of Mood States-Short Form, FACT-HN	Intervention was feasible and acceptable with good patient-compliance. QOL declined over the intervention for the whole population, however, patients with higher post-intervention mindfulness had higher QOL.	4

**Table 3:** Pharmacologic and lifestyle interventions

Study	Settings/Patients	Study Design/Intervention	Timing of intervention	Assessment timepoints	Measures	Results	Methodological Quality
Lydiatt et al. (2013) [24]	148 HNC pts.; Nebraska	RCT. Intervention group received escitalopram pharmacotherapy for 16 weeks vs. placebo pill through their oncology team.	before treatment started	baseline, 2, 4, 6, 8, 10, 12, 16, 20, 24, and 28 weeks	QIDS, MINI, QIDS-C, WQ-QOL, FIBSER (Frequency, Intensity, and Burden of Side Effects Rating)	Prophylactic escitalopram reduced depression rates and improved QOL. Radiation group had highest rates of depression.	6
Capozzi et al. (2016) [25]	60 HNC pts.; Canada	RCT. Intervention group received a lifestyle intervention (physician referral, health education for 6 sessions, behavior change support, individualized exercise program with resistance training, and group exercise setting 2x weekly). Control group was a delayed group of the same intervention.	new diagnosis - randomized between immediate lifestyle intervention vs. 12 week delayed	baseline, 12, 24, 36, 48 weeks	physical activity, BMI, lean body mass and percentage body fat; QOL: FACT-HN; Depression: CES-D, Nutrition - patient generated subjective global assessment	Delayed group were more able to complete intervention. No difference in body composition between groups. Potential benefit regardless of timing. No diff in QOL or depression - both declined at 12 weeks then improved at 24 weeks.	5

**Table 4:** Health Systems interventions

Study	Settings/Patients	Study Design/Intervention	Timing of intervention	Assessment timepoints	Measures	Results	Methodological Quality
van den Brink et al. (2007) [26]	145 HNC pts. in control group, 39 in intervention group; Netherlands	Prospective, non-randomized control trial (intervention vs control based on location). Intervention group given electronic health information support (laptop for communication with healthcare team, information, patient forum, and home symptom monitoring).	post-operatively × 6 weeks; all used intervention	baseline at discharge, 6 weeks, 12 weeks	QOL: Custom questionnaires contained 22 QoL subscales, of which 19 validated in prior studies; usage statistics	All used system. At 6 weeks, intervention arm had improved QOL in 5 of 22 measured parameters. At 12 weeks, only 1 parameter remained significant.	6
D’Souza et al. (2013) [27]	96 HNC pts.; Canada	Non-randomized controlled trial. Intervention group received Multimode Comprehensive Tailored Information Package by treating clinicians (booklet, interactive computer booth, computer animation, DVD, database) at one center and usual info at control center	newly diagnosed HNC, not yet treated	baseline (after information provision but before treatment started), 3 and 6 months post	HADS	Intervention group had significant improvement in anxiety; depression was not as impacted	6

#### Nurse-led interventions

Four studies summarized in Table 1 evaluated interventions that were delivered by nurses [16–19]. Katz et al implemented an educational intervention in which 19 patients were randomized to usual care versus receipt of an educational booklet outlining information on diagnosis, treatment, and coping strategies for patients undergoing surgery for oral cavity cancer, as well as two educational sessions with a nurse [16]. As compared with control patients, patients in the experimental group demonstrated significantly lower anxiety and higher knowledge at the three-month follow-up. There were no differences between the groups for QOL or depression. In a larger study by van der Meulen et al [19], 205 patients were randomized to receive either usual care or the psychological intervention NUCAI (nurse counseling and intervention). NUCAI consisted of six bimonthly sessions on problem-focused counseling to address physical, psychological, and social consequences of HNC and treatment. The patients receiving the intervention reported lower levels of depression 1 year after HNC treatment compared to control patients, as well as decreased physical symptoms including pain and swallowing troubles.

The remaining two studies involved patients who screened positive for psychological dysfunction. Duffy and colleagues randomized patients who screened positive for tobacco use, alcohol use, and/or depression to usual care versus a nurse-administered cognitive behavioral therapy (CBT) intervention with medications when necessary [17]. There were no differences between intervention and control groups in depressive symptoms or alcohol use, both of which decreased during the course of the study across groups. However, patients in the intervention group were more likely to quit smoking than in the usual care group. Finally, Semple and colleagues conducted a non-randomized, controlled study of patients who screened positive for psychological dysfunction of an individualized, psychosocial intervention program delivered at home versus usual care for patients who did not want to participate (control group) [18]. Patients in the intervention group received between 2 and 6 home sessions lasting approximately 90 min each, which targeted pertinent social, psychological, and physical problems faced by patients after treatment. Patients who received the intervention had improvements in anxiety and depression, as well as improved components of QOL. Control patients had no significant improvements in psychological function or QOL over the study time period.

These data suggest that a variety of nurse-led psychosocial or educational interventions improve some aspects of mood and QOL for patients with HNC, but not consistently. The interventions themselves were markedly different, as were the outcome measures used, which makes it challenging to draw unifying conclusions or do cross intervention comparisons. However, it is important to note that patients found the intervention content acceptable, and the nurse-led delivery format was both feasible and acceptable. Patients may be more willing to participate in an intervention that is delivered by someone already involved in their care, such as a nurse, who is without the stigma of being a mental health provider (i.e. a psychologist or psychiatrist). Involving someone already on the care team also would aid in dissemination into the health system. Additionally, the problem focused intervention and the NUCAI intervention both showed significant improvements in patients’ depression, which were their primary outcomes, underscoring that interventions targeting mood in HNC can be effective compared to usual care.

There are important methodological limitations to these studies, however, that have implications on how we interpret the outcomes reported. The only study that reported race was majority white. Therefore, outcomes in a more heterogeneous population are unknown. Two of the studies [16, 18] had small sample sizes with important baseline differences between the control and intervention groups that could account for differences noted in the outcomes. For example, more patients in the control group in Katz et al had more extensive surgeries than those in the experimental group, which could account for the poorer outcomes in the control group because of the greater morbidity of the surgery. In the Semple study [18], the experimental group reported higher levels of anxiety and depression than the control group, and the control group was self-selected for those who did not want to participate in the home intervention. These differences could account for the more pronounced improvements seen in the experimental group, making the effect of the intervention less clear.

Additionally, each intervention was complex and no study analyzed which aspects of the intervention were most helpful. Finally, the interventions were all conducted at different times during patients’ cancer treatment course – one prior to any treatment, two post-treatment, and one involving patients at any time – which can impact how the data are interpreted since psychological and physical symptoms worsen during treatment. Understanding the expected trajectory of mood and QOL throughout treatment is critical to interpreting outcomes. Finally, two of these interventions only enrolled patients who screened positive for psychosocial dysfunction. Thus, application of these interventions to a broader population of patients with HNC is unclear. Interestingly, the NUCAI intervention was a more inclusive population of all patients undergoing treatment for HNC, which showed decreases in depression across the entire study population indicating that benefits can be seen regardless of baseline mood. Overall, it appears that nurse-led interventions targeting physical and psychological functioning are beneficial, though outcomes must be interpreted with caution.

#### Psychologist-led interventions

Six studies in Table 2 employed interventions targeting psychological distress which were delivered by psychologists [20–23, 28]. Hammerlid et al enrolled two small, single arm studies of patients – one group in a long-term group psychological therapy and the second in a week long, short-term psychoeducational program with their caregivers [20]. Patients in both intervention groups received supportive therapy in a group format and reported improvement in emotional, psychosocial, and depressive symptoms. The second study by Allison et al involved a feasibility study with the Nucare coping strategies program, which was a program to teach individuals how to cope with their cancer diagnosis and treatments by way of problem solving, relaxation training, and individualized goal setting either in a group or one-on-one setting with a therapist or at home without a therapist [21]. Following the intervention, patients reported higher QOL and lower depressive symptoms than at baseline. The third study by Kilbourn et al was also a single arm feasibility study that assessed the intervention Easing and Alleviating Symptoms during Treatment (EASE) [28]. All participants received telephone counseling sessions focused on coping and stress management throughout radiation treatment. The intervention was feasible and acceptable, however, baseline to post-intervention QOL scores decreased slightly and pain scores did not change. The fourth study by Pollard et al was also a single arm feasibility study that assessed a mindfulness intervention for patients receiving radiation treatment [29]. This intervention was also feasible and acceptable, but baseline to post-intervention QOL scores decreased across the study timepoints. However, participants with higher post-intervention mindfulness did have higher QOL scores.

The remaining two studies targeted patients with elevated distress. Kangas and colleagues conducted a pilot RCT of an early CBT intervention among 35 patients with elevated levels of PTSD, depression, or anxiety during radiation treatment for HNC [22]. Patients were randomized to receive seven weekly sessions with a clinical psychologist of either a multi-modal cognitive behavioral therapy (CBT) intervention or non-directive supportive counseling (SC). Both interventions equally reduced PTSD and anxiety, however, more patients in the CBT arm no longer met criteria for PTSD, anxiety, and/or depression at the 12- month time point compared to patients receiving SC. The final study was an RCT of a stepped care intervention targeting psychological distress in patients with HNC who screened positive for distress [23]. Patients were randomized to usual care or to the stepped care intervention. Stepped care consisted of watchful waiting, guided self-help, problem-solving therapy, and/or psychotherapy with or without medication, where care would be escalated to the next step if distress did not improve. Patients who received the intervention had greater improvements in symptoms of anxiety and depression compared to patients receiving usual care.

Four of these six studies demonstrate that psychologist-led interventions targeting psychological symptoms in patients with HNC can improve depression and anxiety symptoms, PTSD, and/or QOL. Together, these studies suggest that patients are likely to benefit from programs led by mental health professionals and highlight that they will participate in and value these types of interventions. For example, in Kangas et al, enrollment rate to the study was high (> 80% of those eligible enrolled), and > 70% of participants attended all counseling sessions during their HNC treatment [22]. The results from two of these studies were also more methodologically robust because they used a randomized, controlled design [22, 23], which is more able to assess preliminary efficacy. The positive outcomes across four of the studies also suggest that HNC patients with or without baseline distress can benefit from such interventions since two of the trials included patients with HNC.

These studies also have important methodological limitations. Four had small sample sizes [20, 21, 28] and were single arm studies with no control group, and while Kangas et al was a randomized trial [22], both arms received an intervention with a psychologist, and thus there was no true control. Therefore, the improvements in outcomes noted in these studies could be attributed to a natural improvement over time in QOL and/or psychological symptoms, which is known to occur post-HNC treatment. On the opposite spectrum, the two studies [28, 29] that did not show patient improvement only assessed timepoints at baseline and immediately post-intervention, when participants likely still had significant treatment side effects and thus an improvement of QOL was unlikely to be found. No study reported race or ethnicity. Additionally, there were significant amounts of missing data across the studies, which may have influenced the results. Most of the interventions were also time and likely cost intensive, which potentially limits how well they can be disseminated. Finally, although psychologists have appropriate expertise to deliver these interventions, barriers exist for patients to receive psychological care, which can thereby restrict adoption of the interventions despite known benefits.

#### Pharmacologic intervention

Only one study targeted depression in HNC treatment using a pharmacologic intervention, summarized in Table 3 [24]. Lydiatt et al conducted a randomized, double blind study with prophylactic escitalopram, a selective serotonin reuptake inhibitor approved to treat depression, versus placebo in non-depressed patients undergoing treatment for HNC [24]. The head and neck surgeon involved in the patient’s care gave the overview of the study and a delayed consent process was used. All patients were followed for the study in the surgery clinic. The rate of depression in patients receiving escitalopram was significantly lower than the rate in those receiving placebo, and the QOL of patients receiving escitalopram was significantly higher. This was the first trial in a cancer population to show success in the prevention of depression, and the effect was most pronounced in those receiving radiation therapy where depression was highest. Although there were improved outcomes in both depression and QOL, the concerns of the study revolved around the cost of drug therapy, as well as the possible side effects, though the vast majority of participants reported adverse effects 25% of the time or less. Additionally, the population was ethnically and racially homogenous; thus, its application to a more heterogeneous population remains an important question. Further exploration of which HNC patients may benefit most from this intervention will be important.

#### Lifestyle intervention

One study, also summarized in Table 3, evaluated QOL and mood as a secondary outcome through a lifestyle intervention targeting physical activity and health education [25]. Capozzi et al conducted an RCT of an exercise intervention in patients with newly diagnosed HNC. The intervention included a physician referral and clinic support, health education sessions, and an individual as well as group exercise program with an exercise physiologist and certified personal trainer. Patients were randomized to the 12-week exercise and lifestyle intervention or a wait-list control group, which received the same intervention delayed by 12 weeks. There were no differences between groups in body composition, QOL, or mood at 12 or 24 weeks. It should be noted that patients randomized to the wait-list control (i.e. delayed intervention group) showed greater adherence to the exercise and lifestyle intervention than patients who received the intervention at study onset. Although this study did not show a difference in the primary or secondary outcomes, every participant eventually received the intervention. Thus, there was no true control group for long-term outcomes of 36–48 weeks, which is the time when a positive impact of the intervention may have become clearer. Thus, exploration of this intervention on long-term outcomes as the primary outcome is warranted.

#### System-based interventions

Two other studies, in Table 4, evaluated interventions targeting QOL or mood through a health-system based intervention with a telemedicine system [26] or a tailored information package [27]. Van den Brink, et al led a non-randomized, controlled trial evaluating a telemedicine system integrated into patients’ care compared with a group of patients in a different hospital without access to the telemedicine system [26]. The intervention included a laptop for communication with the health care team, health information, a forum with patients, and home symptom monitoring. Patients in the intervention group had improvements in QOL at 6 weeks post-intervention compared to the control group, though these differences were no longer significant at 12 weeks. The second study by D’Souza et al was also a non-randomized, controlled trial integrating the delivery of a comprehensive information package into patient care compared to a control group at a different hospital who did not receive the information [27]. Patients who received the intervention reported less anxiety than the control group, while there was no difference in depression.

Although these studies are rather different in the interventions tested, each targets QOL and/or mood using novel strategies. There were mild intervention effects across the studies with possible improvements in QOL and anxiety, but both had notable methodological shortcomings. Both were non-randomized, controlled studies and thus baseline differences may have accounted for differences in outcomes. Additionally, the D’Souza study [27] measured baseline data after the intervention had started, which is problematic because the baseline data could have been influenced by the intervention. Finally, the timing of the outcome measures across the study may contribute, in part, to some of the negative results observed. For example, van den Brink [26] only measured outcomes through week 12, which would have been at the end of adjuvant treatment for any patients receiving radiation, a time point when QOL and mood are nadiring. This could explain why the initial differences in QOL between intervention and control groups were no longer evident. Later timepoints when patients have recovered from treatment fully are helpful to assess the long-term effects of the intervention. Given these methodological issues, the negative results of these trials should be interpreted cautiously.

## Discussion

All 14 studies reviewed here used distinct, novel interventions to improve QOL and mood in patients with HNC. The studies included different HNC patient populations, cancer stages, follow-up times, outcome measures, and were of variable quality. However, despite these differences, the studies illustrate that these interventions are well-received by patients and can be beneficial to them. The majority of the interventions led to a positive impact on patient-reported QOL and/or mood. Although determining which intervention was the most successful is difficult, important themes emerged from the interventions as a whole. First, nurse-led and psychologist-led interventions were both successful in improving patient outcomes, but nurse-led interventions may be easier to integrate into patients’ care with less stigma and higher availability, in particular when the intervention visits are coordinated with other treatment visits for their cancer or at the patients’ homes. Second, education was a critical component of many of the interventions, underscoring the importance of patients’ understanding of the cancer and treatment, and how improved understanding can positively impact QOL and/or mood. Third, most intervention components involved several sessions, emphasizing that ongoing reinforcement of the topics for patients is necessary to effect change. Fourth, multicomponent interventions were important because they were able to address multiple aspects of patient care simultaneously in order to impact QOL and/or mood. Finally, many of these intervention trials included all patients with HNC and showed positive effects across the entire population studied. Therefore, these types of interventions should not only be considered for those who are already distressed, but rather for the HNC population as a whole given the significant burden of treatment.

### Challenges and future directions

The studies reviewed also highlight significant limitations of the research methodology employed that must be understood in order to improve future studies. First, many of these studies were either single arm studies, or had small sample sizes as seen in the quality assessment, limiting conclusions that can be drawn about efficacy. Necessary next steps will involve conducting larger RCTs, which can appropriately assess efficacy. Second, there was inconsistency across these studies in the outcome measures used to assess QOL and/or mood, as well as in the timing of outcome assessments. Future studies should be standardized in the outcome measures used as well as the timing of the measurement in order to be able to draw consistent conclusions. Ideally, patients should be enrolled at the same point in time in relation to their treatment (either prior to treatment start or at a specified point post-treatment) and then assessments should be measured at baseline, post treatment if applicable, and at 3, 6, and 12 months due to the impact of treatment and length of recovery. Including a mixed population is particularly problematic in HNC because patients in active treatment have worse QOL and mood than those before or after treatment. Thus, a mixed population of patients may introduce major differences in outcome data that could be misinterpreted as an intervention effect. Third, only 4 of the 14 studies measured long-term outcomes at 1 year or later, which is an important timepoint in patients’ treatment trajectories in regard to long-term QOL and mood. Many studies also had significant drop off in response rates of the outcomes on later assessments, which could have biased results. Future studies should enroll patients at the same point in time in their treatment trajectory in order to minimize baseline differences, include long-term outcome measures, and maintain high response rates to the extent possible such that the data is representative of the population.

The majority of interventions were led by a healthcare professional (nurse, psychologist, clinician, exercise physiologist), which can be labor- or cost-intensive. However, recent technological advances have resulted in the development of novel digital health interventions that have the potential to positively benefit cancer survivors [30]. Although few such interventions have been evaluated in patients with HNC, one study found that oral cancer survivors rated a web-based symptom management program favorably and were interested in using online tools to improve QOL [31]. Thus, a combined program that integrates online resources with in-person support may be helpful in promoting positive psychological outcomes at reduced costs.

Only two of the interventions were focused on the healthcare delivery system in which patients were receiving care, and no intervention was focused on the dissemination and implementation of the intervention into the healthcare system. In order to improve health outcomes in patients with HNC on a population level, determining how to implement these interventions health system-wide will be critical to establishing feasibility.

Finally, this review focused primarily on interventions for patients with HNC. However, caregivers of these patients also report significant distress and poor QOL during the patient’s treatment, which can also impact outcomes [15]. There are even fewer interventions targeting caregivers or caregiver and patient dyads in HNC. Thus, broadening future interventions to include a caregiver component will also be important to improve overall outcomes.

In conclusion, with the small number of studies meeting criteria for our review, it is clear that further research in interventions focusing on improving mood and QOL in patients with HNC is needed. Although many of these studies had positive outcomes, more than half had sample sizes of 60 of fewer patients, or were pilot in nature. In order to determine what interventions are most beneficial to our patients, we must move beyond pilot studies to well-designed, rigorous, RCTs that are adequately powered to detect meaningful improvements in short and long-term QOL and mood. We must also include caregivers in these studies where applicable. Once efficacy is clear, we must then determine how to best implement and disseminate the interventions health system-wide in order to reduce the significant physical and psychological burden of HNC for our patients and their caregivers.

## Data Availability

Not applicable.

## Abbreviations

CBT: Cognitive behavioral therapy
CES-D: Center for Epidemiological Studies – Depression Score
EASE: Easing and Alleviating Symptoms during Treatment
EORTC: European Organization of Research and Treatment of Cancer Therapy
FACT: Functional Assessment of Cancer Therapy
HADS: Hospital Anxiety and Depression Scale
HNC: Head and neck cancer
NUCAI: Nurse counseling and intervention
PTSD: Post-traumatic stress disorder
QIDS: Quick Inventory of Depressive Symptomatology
QOL: Quality of life
RCT: Rrandomized control trial
SC: Supportive counseling
UWQOL: University of Washington Quality of Life

## References

[1] Devins GM, Payne AY, Lebel S, Mah K, Lee RN, Irish J (2013). The burden of stress in head and neck cancer. Psychooncology..

[2] Gunn GB, Mendoza TR, Fuller CD, Gning I, Frank SJ, Beadle BM (2013). High symptom burden prior to radiation therapy for head and neck cancer: a patient-reported outcomes study. Head Neck.

[3] Murphy BA, Ridner S, Wells N, Dietrich M (2007). Quality of life research in head and neck cancer: a review of the current state of the science. Crit Rev Oncol Hematol.

[4] Gil F, Costa G, Hilker I, Benito L (2012). First anxiety, afterwards depression: psychological distress in cancer patients at diagnosis and after medical treatment. Stress Health.

[5] Lydiatt WM, Moran J, Burke WJ (2009). A review of depression in the head and neck cancer patient. Clin Adv Hematol Oncol.

[6] Astrup GL, Rustoen T, Miaskowski C, Paul SM, Bjordal K (2015). A longitudinal study of depressive symptoms in patients with head and neck Cancer undergoing radiotherapy. Cancer Nurs.

[7] Badr H, Gupta V, Sikora A, Posner M (2014). Psychological distress in patients and caregivers over the course of radiotherapy for head and neck cancer. Oral Oncol.

[8] Neilson K, Pollard A, Boonzaier A, Corry J, Castle D, Smith D (2013). A longitudinal study of distress (depression and anxiety) up to 18 months after radiotherapy for head and neck cancer. Psychooncology..

[9] Kim SA, Roh JL, Lee SA, Lee SW, Kim SB, Choi SH (2016). Pretreatment depression as a prognostic indicator of survival and nutritional status in patients with head and neck cancer. Cancer..

[10] Barber B, Dergousoff J, Slater L, Harris J, O'Connell D, El-Hakim H (2016). Depression and survival in patients with head and neck Cancer: a systematic review. JAMA Otolaryngol Head Neck Surg.

[11] Posluszny DM, Dougall AL, Johnson JT, Argiris A, Ferris RL, Baum A (2015). Posttraumatic stress disorder symptoms in newly diagnosed patients with head and neck cancer and their partners. Head Neck..

[12] Moschopoulou E, Hutchison I, Bhui K, Korszun A (2018). Post-traumatic stress in head and neck cancer survivors and their partners. Support Care Cancer.

[13] Vickery LE, Latchford G, Hewison J, Bellew M, Feber T (2003). The impact of head and neck cancer and facial disfigurement on the quality of life of patients and their partners. Head Neck..

[14] Sanjida S, McPhail SM, Shaw J, Couper J, Kissane D, Price MA (2018). Are psychological interventions effective on anxiety in cancer patients? A systematic review and meta-analyses. Psychooncology..

[15] Longacre ML, Ridge JA, Burtness BA, Galloway TJ, Fang CY (2012). Psychological functioning of caregivers for head and neck cancer patients. Oral Oncol.

[16] Katz MR, Irish JC, Devins GM (2004). Development and pilot testing of a psychoeducational intervention for oral cancer patients. Psychooncology..

[17] Duffy SA, Ronis DL, Valenstein M, Lambert MT, Fowler KE, Gregory L (2006). A tailored smoking, alcohol, and depression intervention for head and neck cancer patients. Cancer Epidemiol Biomark Prev.

[18] Semple C, Parahoo K, Norman A, McCaughan E, Humphris G, Mills M. Psychosocial interventions for patients with head and neck cancer. Cochrane Database Syst Rev. 16(2013, 7):CD009441. 10.1002/14651858.CD009441.pub2 PMC11936101 23857592

[19] van der Meulen IC, May AM, de Leeuw JR, Koole R, Oosterom M, Hordijk GJ (2014). Long-term effect of a nurse-led psychosocial intervention on health-related quality of life in patients with head and neck cancer: a randomised controlled trial. Br J Cancer.

[20] Hammerlid E, Persson LO, Sullivan M, Westin T (1999). Quality-of-life effects of psychosocial intervention in patients with head and neck cancer. Otolaryngol Head Neck Surg.

[21] Allison PJ, Nicolau B, Edgar L, Archer J, Black M, Hier M (2004). Teaching head and neck cancer patients coping strategies: results of a feasibility study. Oral Oncol.

[22] Kangas M, Milross C, Taylor A, Bryant RA (2013). A pilot randomized controlled trial of a brief early intervention for reducing posttraumatic stress disorder, anxiety and depressive symptoms in newly diagnosed head and neck cancer patients. Psychooncology..

[23] Krebber AM, Buffart LM, Kleijn G, Riepma IC, de Bree R, Leemans CR (2014). Prevalence of depression in cancer patients: a meta-analysis of diagnostic interviews and self-report instruments. Psychooncology..

[24] Lydiatt WM, Bessette D, Schmid KK, Sayles H, Burke WJ (2013). Prevention of depression with escitalopram in patients undergoing treatment for head and neck cancer: randomized, double-blind, placebo-controlled clinical trial. JAMA Otolaryngol Head Neck Surg..

[25] Capozzi LC, McNeely ML, Lau HY, Reimer RA, Giese-Davis J, Fung TS (2016). Patient-reported outcomes, body composition, and nutrition status in patients with head and neck cancer: results from an exploratory randomized controlled exercise trial. Cancer..

[26] van den Brink JL, Moorman PW, de Boer MF, Hop WC, Pruyn JF, Verwoerd CD (2007). Impact on quality of life of a telemedicine system supporting head and neck cancer patients: a controlled trial during the postoperative period at home. J Am Med Inform Assoc.

[27] D'Souza V, Blouin E, Zeitouni A, Muller K, Allison PJ (2013). An investigation of the effect of tailored information on symptoms of anxiety and depression in head and neck cancer patients. Oral Oncol.

[28] Kilbourn KM, Anderson D, Costenaro A, Lusczakoski K, Borrayo E, Raben D (2013). Feasibility of EASE: a psychosocial program to improve symptom management in head and neck cancer patients. Support Care Cancer.

[29] Pollard A., Burchell J.L., Castle D., Neilson K., Ftanou M., Corry J., Rischin D., Kissane D.W., Krishnasamy M., Carlson L.E., Couper J. (2016). Individualised mindfulness-based stress reduction for head and neck cancer patients undergoing radiotherapy of curative intent: a descriptive pilot study. European Journal of Cancer Care.

[30] Escriva Boulley G, Leroy T, Bernetiere C, Paquienseguy F, Desfriches-Doria O, Preau M (2018). Digital health interventions to help living with cancer: a systematic review of participants' engagement and psychosocial effects. Psychooncology..

[31] Badr H, Lipnick D, Diefenbach MA, Posner M, Kotz T, Miles B (2016). Development and usability testing of a web-based self-management intervention for oral cancer survivors and their family caregivers. Eur J Cancer Care (Engl)..

